# Effect of organic acids on the growth and lipid accumulation of oleaginous yeast *Trichosporon fermentans*

**DOI:** 10.1186/1754-6834-5-4

**Published:** 2012-01-19

**Authors:** Chao Huang, Hong Wu, Zong-jun Liu, Jun Cai, Wen-yong Lou, Min-hua Zong

**Affiliations:** 1 State Key Laboratory of Pulp and Paper Engineering, College of Light Industry and Food Sciences, South China University of Technology, 381 Wushan Rd.,Tianhe District, Guangzhou 510640, China; 2 School of Biosciences and Bioengineering, South China University of Technology, 382 East Waihuan Rd., Guangzhou Higher Education Mega Centre, Panyu District, Guangzhou 510640, China; 3 Key Laboratory of Fermentation Engineering, Ministry of Education, Hubei University of Technology, 1 Lijiadun Rd., Wuhan 430068, China

**Keywords:** lignocellulosic hydrolysate, organic acid, inhibition, lipid production, *Trichosporon fermentans*

## Abstract

**Background:**

Microbial lipids have drawn increasing attention in recent years as promising raw materials for biodiesel production, and the use of lignocellulosic hydrolysates as carbon sources seems to be a feasible strategy for cost-effective lipid fermentation with oleaginous microorganisms on a large scale. During the hydrolysis of lignocellulosic materials with dilute acid, however, various kinds of inhibitors, especially large amounts of organic acids, will be produced, which substantially decrease the fermentability of lignocellulosic hydrolysates. To overcome the inhibitory effects of organic acids, it is critical to understand their impact on the growth and lipid accumulation of oleaginous microorganisms.

**Results:**

In our present work, we investigated for the first time the effect of ten representative organic acids in lignocellulosic hydrolysates on the growth and lipid accumulation of oleaginous yeast *Trichosporon fermentans *cells. In contrast to previous reports, we found that the toxicity of the organic acids to the cells was not directly related to their hydrophobicity. It is worth noting that most organic acids tested were less toxic than aldehydes to the cells, and some could even stimulate the growth and lipid accumulation at a low concentration. Unlike aldehydes, most binary combinations of organic acids exerted no synergistic inhibitory effects on lipid production. The presence of organic acids decelerated the consumption of glucose, whereas it influenced the utilization of xylose in a different and complicated way. In addition, all the organic acids tested, except furoic acid, inhibited the malic activity of *T. fermentans*. Furthermore, the inhibition of organic acids on cell growth was dependent more on inoculum size, temperature and initial pH than on lipid content.

**Conclusions:**

This work provides some meaningful information about the effect of organic acid in lignocellulosic hydrolysates on the lipid production of oleaginous yeast, which is helpful for optimization of biomass hydrolysis processes, detoxified pretreatment of hydrolysates and lipid production using lignocellulosic materials.

## Background

Biodiesel, a mixture of long-chain monoalkyl fatty acid esters, has been considered a good alternative to conventional petrodiesel oil because of its green and renewable characteristics [1]. Although it has been used in many countries around the world, the high production cost, of which oil feedstock accounts for about 75%, has become a hurdle, and the sustainable and stable supply of cheap lipids is crucial for their further development and wide application [2]. Nowadays, the most commonly used feedstocks in biodiesel production are vegetable oils and waste oils from restaurants or industry. However, vegetable oils such as rapeseed oil and corn oil contribute to the world's food supply, and thus their use as feedstock for biodiesel production has brought about the food versus biofuel debate [3]. The amount of waste oils is limited and cannot meet the increasing demand for biofuel. Microbial oils, namely, single-cell oils (SCOs), which have long been used as substitutes for high-added-value lipids [4,5] such as cocoa butter [6,7], are now believed to be a promising candidate as biodiesel feedstock because of their fatty acid composition, which is similar to those of vegetable oils [8]. At present, however, the high fermentation cost of SCOs limits their use for biodiesel production [7,9]. The adoption of inexpensive media, such as molasses [10], industrial glycerol [11], monosodium glutamate wastewater [2] and lignocellulosic hydrolysates [12] for lipid fermentation is one of the possible resolutions of this problem. Recently, the use of lignocellulosic materials for SCO production has attracted increasing attention because these materials are the most abundant and renewable biomass resources in nature [8,12].

Lignocellulosic biomass consists of cellulose, hemicellulose and lignin, whose relative proportion depends on their material sources [13]. The hydrolysis of lignocellulosic materials into soluble, fermentable sugars is necessary for their efficient utilization by microorganisms. However, a variety of by-products, mainly organic acids, aldehydes and alcohols such as acetic acid, furfural from decomposition of pentoses, 5-hydroxymethylfurfural from degradation of hexoses and aromatics (aromatic alcohols, acids and aldehydes) from lignin, are inevitably generated during hydrolysis with dilute acid [14]. In most cases, these by-products, known as "inhibitors," exert negative effects on the growth, metabolism and product formation of microorganism cells in the fermentation process [15].

Recently, we reported that despite the oleaginous yeast *Trichosporon fermentans*'s production of a poor lipid yield on nondetoxified, sulfuric acid-treated rice straw hydrolysate (SARSH), it grew well with efficient lipid accumulation on detoxified SARSH [12], suggesting that the inhibitors in the lignocellulosic hydrolysate do have great effects on lipid fermentation. Among the inhibitors, organic acids are generally the most abundant, and ten kinds of organic acids, including aliphatic acids (acetic acid, formic acid, levulinic acid and caproic acid), aromatic or furan acids (4-hydroxybenzoic acid, syringic acid, vanillic acid, furoic acid, ferulic acid and gallic acid) have been found in lignocellulosic hydrolysate. Little is known about their inhibition on lipid fermentation, however [16,17]. To provide some interesting information necessary for lipid fermentation on lignocellulosic hydrolysates, we systematically investigated, for the first time, the inhibitory effects of the above-mentioned organic acids on the growth and lipid accumulation of *T. fermentans *with a mixture of glucose and xylose at a ratio of 2:1 (wt/wt) as the carbon source, owing to its similarity to lignocellulosic hydrolysates.

## Results and discussion

**Figure 1:**
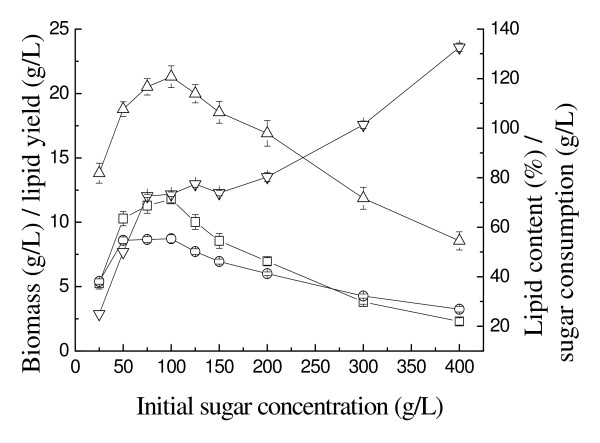
**Effect of initial sugar concentration on the growth and lipid accumulation of *Trichosporon fermentans***. Triangles, biomass; circles, lipid content; squares, lipid yield; inverted triangles, sugar consumption.

All microbial processes are affected by the sugar concentration in the medium, and substrate inhibition may occur during growth of oleaginous microorganisms on sugars [18]. Therefore, we first investigated the effect of sugar concentration, ranging from 25 to 400 g/L, on cell growth, lipid accumulation and sugar consumption of *T. fermentans*. As shown in Figure 1, there was no significant substrate inhibition on the cell growth of *T. fermentans *at concentrations up to 100 g/L, but cell growth was repressed at higher sugar concentrations, especially ≥200 g/L. The highest biomass, lipid content and lipid yield occurred at 100 g/L sugar concentration. At this point, about 73.3 g/L total sugars were utilized by *T. fermentans *after five days' fermentation, and the biomass and lipid yield per sugar consumed were 29.1% and 16.1% (g/g), respectively. Therefore, 100 g/L was chosen as the initial sugar concentration in the subsequent experiments.

**Figure 2:**
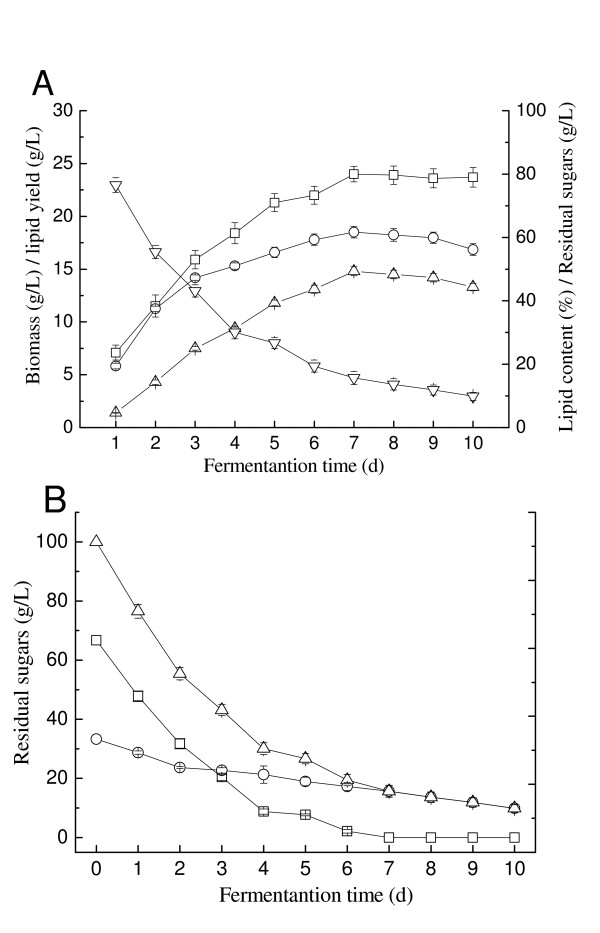
**Production of microbial oils on medium without inhibitors by *Trichosporon fermentans***. **(A) **Time courses of growth and lipid accumulation. Squares, biomass; circles, lipid content; triangles, lipid yield. **(B) **Time course of sugar utilization. Squares, glucose; circles, xylose; triangles, total sugars.

In our previous study, we showed that the optimal inoculum size, temperature and initial pH for lipid production by *T. fermentans *at 100 g/L glucose are 5%, 25°C and 6.5, respectively [10]. In our present work, albeit using a mixture of glucose and xylose (100 g/L) at a ratio of 2:1 (wt/wt) as a carbon source, there was no change in the optimal fermentation conditions (inoculum size, temperature and initial pH), as indicated in Table 1. Figure 2 depicts the time courses of cell growth, lipid accumulation and sugar utilization of *T. fermentans *in the same medium against fermentation time. As shown in Figure 2A, the biomass increased with the increase of fermentation time and reached the maximum on day 7. Further increase in the fermentation time resulted in little variation in biomass production. The highest lipid content and lipid yield were also obtained on the seventh day, on which the SCO produced per total sugar consumed was 17.6% (g/g). Interestingly, *T. fermentans *could utilize glucose and xylose simultaneously (Figure 2B), which would be beneficial for large-scale application because it would shorten the fermentation time.

**Table 1:** Effect of fermentation conditions on the cell growth and lipid accumulation of *Trichosporon fermentans *on the medium without inhibitor

Inoculum size (%, vol/vol)	Initial pH	Temperature (°C)	Biomass (g/L)	Lipid content (%, g/g)	Lipid yield (g/L)
5%	6.5	25	24.0 ± 0.7	61.7 ± 1.6	14.8 ± 0.5
10%	6.5	25	22.4 ± 1.2	58.6 ± 1.7	13.1 ± 1.0
15%	6.5	25	21.6 ± 1.1	54.3 ± 1.2	11.7 ± 0.9
5%	5.5	25	18.4 ± 0.9	57.2 ± 1.7	10.5 ± 0.8
5%	7.5	25	21.5 ± 0.7	56.3 ± 1.4	12.1 ± 0.7
5%	6.5	22	19.9 ± 0.9	55.8 ± 1.1	11.1 ± 0.7
5%	6.5	28	23.6 ± 1.2	58.9 ± 1.2	13.9 ± 1.0

### Effects of organic acids on the growth and lipid accumulation of *T. fermentans*

**Figure 3:**
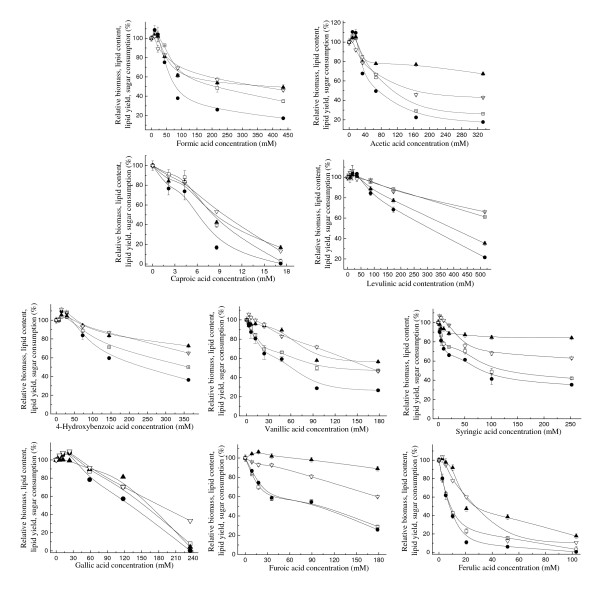
**Effect of selected organic acids on the growth and lipid accumulation of *Trichosporon fermentans***. The biomass, lipid content, lipid yield and sugar consumption of *T. fermentans *on the seventh day in medium without inhibitors were, respectively, 24.0 g/L, 61.7%, 14.8 g/L and 84.3 g/L. Open squares, relative biomass; closed triangles, relative lipid content; closed circles, relative lipid yield; open inverted triangles, relative sugar consumption.

Among the ten kinds of acids tested, caproic and ferulic acids showed the strongest inhibitory effects, and the relative biomass levels of *T. fermentans *were only about 3% and 23% in the presence of 17.2 mM (2 g/L) caproic acid and 20.6 mM (4 g/L) ferulic acid, respectively (Figure 3). Fortunately, caproic and ferulic acids are normally found at very low concentrations in lignocellulosic hydrolysates. For example, in brewer's spent grain and red oak hydrolysates, the concentrations of ferulic acid and caproic acid are only around 0.3 mM (0.06 g/L) and 0.17 mM (0.02 g/L), respectively [19,20], which cause about 4% and 2% inhibition of lipid yield of *T. fermentans*, respectively (Figure 3), and hardly affect the lipid production by *T. fermentans*. The inhibitory effects of the other eight kinds of acids are shown in Figure 3. Similarly, all these organic acids showed only a slight inhibitory effect on the growth and lipid accumulation of *T. fermentans *at their likely concentrations in lignocellulosic hydrolysates [15]. Interestingly, although the inhibition of furoic acid on cell growth became more serious with the increase of its concentration, no significant decrease in the lipid content was observed, even when its concentration reached 89.2 mM (10 g/L), which differs from previous reports that both cell growth and lipid synthesis of oleaginous yeast *Rhodosporidium toruloides *were seriously suppressed by furoic acid, even at concentrations as low as 4 mM [17], demonstrating that the inhibition of an organic acid on lipid fermentation varies widely with different microorganism strains. Among the organic acids tested, levulinic acid exerted the least impact on the lipid yield. It is worth noting that, rather than suppressing cell growth and lipid accumulation, some organic acids, including formic acid, acetic acid, levulinic acid, 4-hydroxybenzoic acid and gallic acid, might even stimulate both cell growth and lipid accumulation of *T. fermentans *when their concentrations are below 16 mM. A similar phenomenon was also observed in that low concentrations of phenol-type compounds (< 5 mM of gallic acid equivalent) stimulated biomass production and citric acid biosynthesis of yeast *Yarrowia lipolytica *when olive mill waste waters were used as a substrate for citric acid production [21]. Also, it has been reported that the biomass and SCO production by another *Y. lipolytica *strain was remarkably enhanced when the medium was supplemented with *Teucrium polium *extract (in quantities of 10 g/L) [22]. More recently, *Y. lipolytica *has been further proven to produce substantially higher SCO quantities in media supplemented with phenol compounds (concentrations ranging from 5 to 10 mM) [23].

IC_25 _and IC_50_, which represent the molar concentrations of the tested organic acids that cause 25% and 50% inhibition of the lipid yield of *T. fermentans*, respectively, are summarized in Table 2. The relative biomass and lipid content at IC_25 _and IC_50 _concentrations are also given in Table 2. Apparently, there is no direct relationship between the toxicity and hydrophobicity of organic acid, which does not agree with the previous report describing ethnologenic *Escherichia coli*, in which the investigators found that the more hydrophobic the organic acid, the stronger its inhibitory effect [24]. In most cases, the molar concentrations of IC_25 _and IC_50 _for organic acids are higher than the corresponding values for aldehydes [25]. For example, the IC_50 _of vanillic acid was 60.7 mM but 6.6 mM for vanillin, suggesting that organic acids are less toxic than aldehydes to lipid production of *T. fermentans*.

**Table 2:** Concentration of organic acids required to inhibit the lipid yield of *Trichosporon fermentans*

Organic acid	IC_25_^a^ (mM)	Relative biomass (%)	Relative lipid content (%)	IC_50_^b^ (mM)	Relative biomass (%)	Relative lipid content (%)	LogP^c^
Formic acid	43.4	92	81	80.4	72	68	-0.54
Acetic acid	30.0	87	83	66.6	68	78	-0.17
Levulinic acid	146.4	90	82	310.0	79	61	-0.49
4-hydroxybenzoic acid	97.7	84	89	188.2	67	80	1.58
Vanillic acid	15.5	80	93	60.7	63	79	1.43
Syringic acid	9.6	79	94	78.2	58	86	1.04
Furoic acid	17.8	70	106	93.7	52	98	1.06
Gallic acid	67.6	88	86	135.2	63	74	0.7
Ferulic acid	3.1	76	99	7.7	51	97	1.51
Caproic acid	3.0	88	83	5.6	71	69	1.92

^a^Molar concentration of 25% inhibition on lipid yield. ^b^Molar concentration of 50% inhibition on lipid yield. ^c^The LogP data are from work by Zaldivar and Ingram [24].

The sugar consumption in the medium containing the selected organic acid was also recorded after fermentation for seven days at which the control without inhibitor gave the maximum lipid yield and the residual sugar was xylose with a concentration of about 15.7 g/L (Figure 3). Interestingly, except for furoic and caproic acids, the relative sugar consumption was above 100% in the presence of a small amount of organic acids. However, improved sugar utilization did not necessarily lead to an enhanced lipid yield. For example, *T. fermentans *could utilize more sugars than the control in the presence of 5 mM syringic acid, but the corresponding relative lipid yield was only 81.1%. At higher concentrations, however, all the tested organic acids suppressed the sugar utilization, and the higher the concentration, the more pronounced the suppression. Among the organic acids examined, levulinic acid showed the least influence on sugar utilization, which is in accordance with the observation that levulinic acid displayed the lowest toxicity to lipid production of *T. fermentans*.

**Figure 4:**
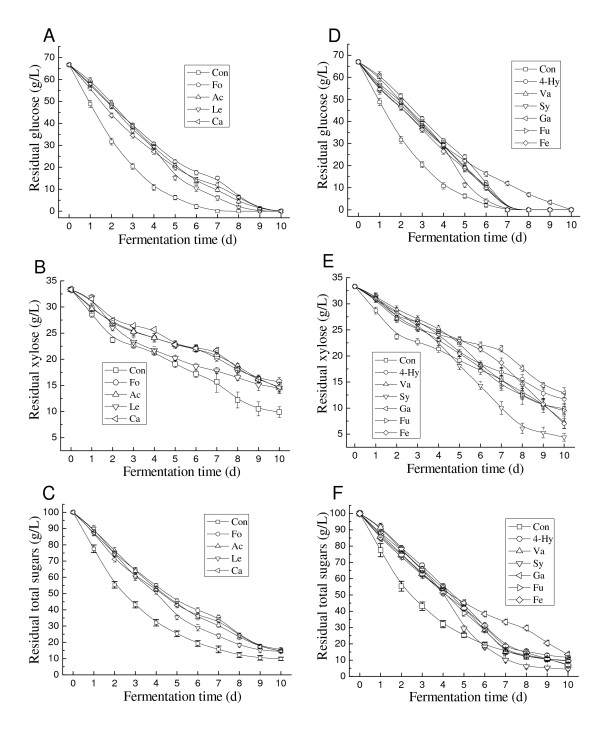
**Effect of the selected organic acids on the sugar metabolism of *Trichosporon fermentans***. **(A) **and **(D) **Glucose consumption. **(B) **and **(E) **Xylose consumption. **(C) **and **(F) **Total sugar consumption. Ac, acetic acid; Fo, formic acid; Le, levulinic acid; Hy, 4-hydroxybenzoic acid; Fu, furoic acid; Ca, caproic acid; Ga, gallic acid; Fe, ferulic acid; Sy, syringic acid; Va, vanillic acid.

To gain deeper insight into the sugar assimilation, the time course of sugar utilization in the medium containing the selected organic acid at its IC_25 _was further investigated. As shown in Figures 4A and 4D, the glucose consumption rate decreased in the presence of acids, but it still could be exhausted by *T. fermentans *with an increase in time, as reported by Narendranath *et al*. [26] and Huang *et al*. [12]. Similarly, aliphatic acids decreased the xylose consumption rate throughout the fermentation process, and less xylose was utilized compared with the control (Figure 4B). In the presence of aromatic or furan acids such as vanillic, syringic, furoic and ferulic acids, however, *T. fermentans *can utilize xylose at a faster rate than the control from day 5, and more xylose was consumed at the end of fermentation (Figure 4E). Obviously, in some cases, organic acids can stimulate the utilization of xylose. However, the enhanced sugar conversion did not result in improved lipid production. A similar phenomenon was also observed in our previous work on the effect of aldehyde on the growth and lipid accumulation of *T. fermentans *[25]. The reason for this is now under investigation in our laboratory.

In our previous studies [10,12], we found that when glucose is almost exhausted, cellular lipids can be used as a carbon source to maintain the growth of *T. fermentans*. In general, microorganisms consume their accumulated lipids mainly through the glyoxylate bypass pathway, and, more specifically, different microbes might preferentially consume different kinds of fatty acids to maintain their growth [6]. In this work, there is also apparent cellular lipid degradation between the seventh and tenth days for control without inhibitors. A similar tendency was observed in the culture of *T. fermentans *on the media containing various organic acids (data not shown). The lipid degradation rate is slower than the control, however, because of the presence of acids, especially the aromatic acids, suggesting that organic acids repress lipid turnover as well. This is an interesting phenomenon because repression of accumulated lipid degradation has been observed only in multiple limited media [27].

**Figure 5:**
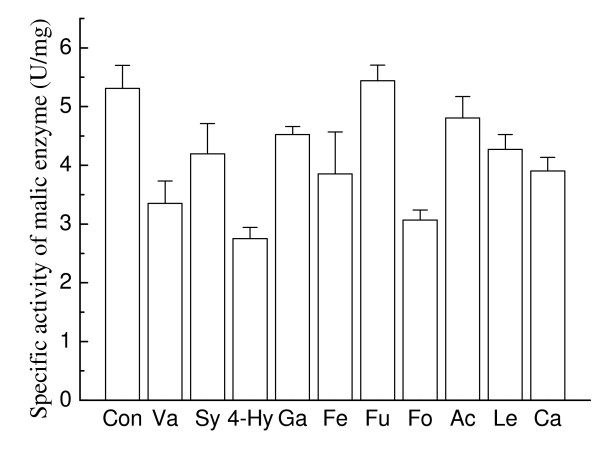
**Effects of selected organic acids on malic enzyme activity in *Trichosporon fermentans***. Con, control; Ac, acetic acid; Fo, formic acid; Le, levulinic acid; Hy, 4-hydroxybenzoic acid; Fu, furoic acid; Ca, caproic acid; Ga, gallic acid; Fe, ferulic acid; Sy, syringic acid; Va, vanillic acid.

Malic enzyme is considered the key enzyme for lipid synthesis in oleaginous microorganisms because it is one of the main enzymes providing a supply of NADPH (nicotinamide adenine dinucleotide phosphate oxidase) to microorganisms [28,29]. In this work, the effects of the selected organic acids (each at its IC_25 _concentration) on the malic enzyme activity were tested after the second day of fermentation, when the lipid formation rate reached the maximum in culture without an inhibitor. As shown in Figure 5, the malic enzyme activity was inhibited by all the organic acids tested except furoic acid, which can well explain the delay in lipid accumulation in the media containing these acids. Interestingly, for furoic acid, there was no significant change in the specific activity of malic enzyme, and even a higher malic enzyme activity was detected, which partly explains the small influence of furoic acid on the lipid synthesis of *T. fermentans *mentioned above (Figure 3).

In many cases, addition of natural compounds, such as sterculia oil [4,5], essential oils [30], plant extracts [31] or phenol-type compounds [20], even in small concentrations, can have profound effects upon the fatty acid composition of microbial lipids. High-added-value microbial lipids such as cocoa butter substitutes can be produced by modifying the fatty acid composition of lipids under various conditions [4,5]. The effect of organic acids on the fatty acid composition of lipids from *T. fermentans *is shown in Table 3. Apparently, oleic acid was the most abundant fatty acid, being about 50% to 60% of the total fatty acids, followed by palmitic acid, stearic acid and linoleic acid. In most cases, organic acid had no significant influence on the composition of unsaturated acids, including oleic acid and linoleic acid. However, the presence of organic acid would increase the palmitic acid content. The stearic acid content varied with the organic acid. It is worth noting that acetic acid, the most abundant acid in the lignocellulosic hydrolysates, had little influence on the fatty acid composition of lipid produced by *T. fermentans*.

**Table 3:** Effect of organic acids on the fatty acid composition of lipids

	Fatty acid composition (%)				
	
Organic acid^a^	Palmitic acid (C16:0)	Linoleic acid (C18:2)	Oleic acid (C18:1)	Stearic acid (C18:0)	Others
Acetic acid (2 g/L)	19.8	6.6	55.9	16.5	1.2
Syringic acid (0.5 g/L)	22.9	5.9	47.6	22.6	1
Vanillic acid (0.5 g/L)	23.6	5.8	47.1	22.5	1
Caproic acid (0.25 g/L)	24	6.8	56.9	10.9	1.4
Furoic acid (0.5 g/L)	26.1	5.6	53	14.4	0.9
4-hydroxybenzoic acid (0.5 g/L)	21.2	5.8	54.1	17.9	1
Formic acid (1 g/L)	19.5	6.6	57.8	15.2	0.9
Levulinic acid (4 g/L)	22.6	5.6	54.2	15.8	1.8
Ferulic acid (2 g/L)	20.7	4.8	60.4	11.8	2.3
Gallic acid (4 g/L)	17.2	7.3	56.6	18.3	0.6
Control	17.4	6.1	57.9	17.4	1.2

^a^Values in parentheses represent the organic acid's concentration tested.

### Effects of inoculum size, temperature, and initial pH on the inhibition by organic acids

**Figure 6:**
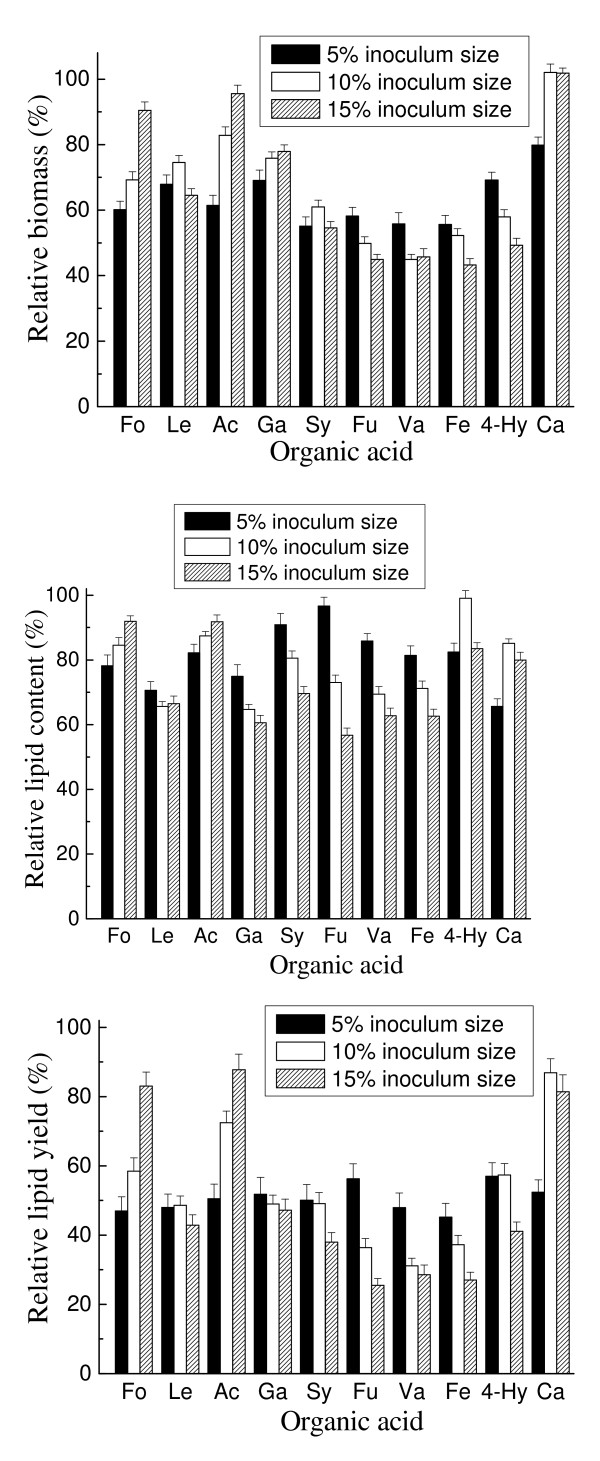
**Effect of inoculum size on the inhibition of organic acids**. Each organic acid was tested at the molar concentration of the tested organic acids that caused 50% inhibition of the lipid yield of *Trichosporon fermentans*. Cultures were incubated initially at pH 6.5, 25°C and 160 rpm for 7 days. The results are expressed relative to controls without organic acids. Biomass, lipid content and lipid yield of cultures in the absence of organic acids with 5%, 10% and 15% inoculum sizes were, respectively, 24.0 g/L, 61.7% and 14.8 g/L; 22.4 g/L, 58.6% and 13.1 g/L; and 21.6 g/L, 54.3% and 11.7 g/L. Ac, acetic acid; Fo, formic acid; Le, levulinic acid; Hy, 4-hydroxybenzoic acid; Fu, furoic acid; Ca, caproic acid; Ga, gallic acid; Fe, ferulic acid; Sy, syringic acid; Va, vanillic acid.

It has been reported that the toxicity of organic acids to microorganism can be relieved by increasing cell density in fermentation [24]. Therefore, each selected organic acid was added to the medium at its IC_50 _concentration to examine the effect of inoculum size on the inhibition of the organic acid (Figure 6). In most cases, increasing inoculum size cannot reduce the inhibitory effects of acids on cell growth and lipid accumulation. Unexpectedly, in the case of caproic acid, high inoculum size (10% and 15%) not only eliminated its inhibition on cell growth but also enhanced cell growth, as indicated by a relative biomass of more than 100%.

**Figure 7:**
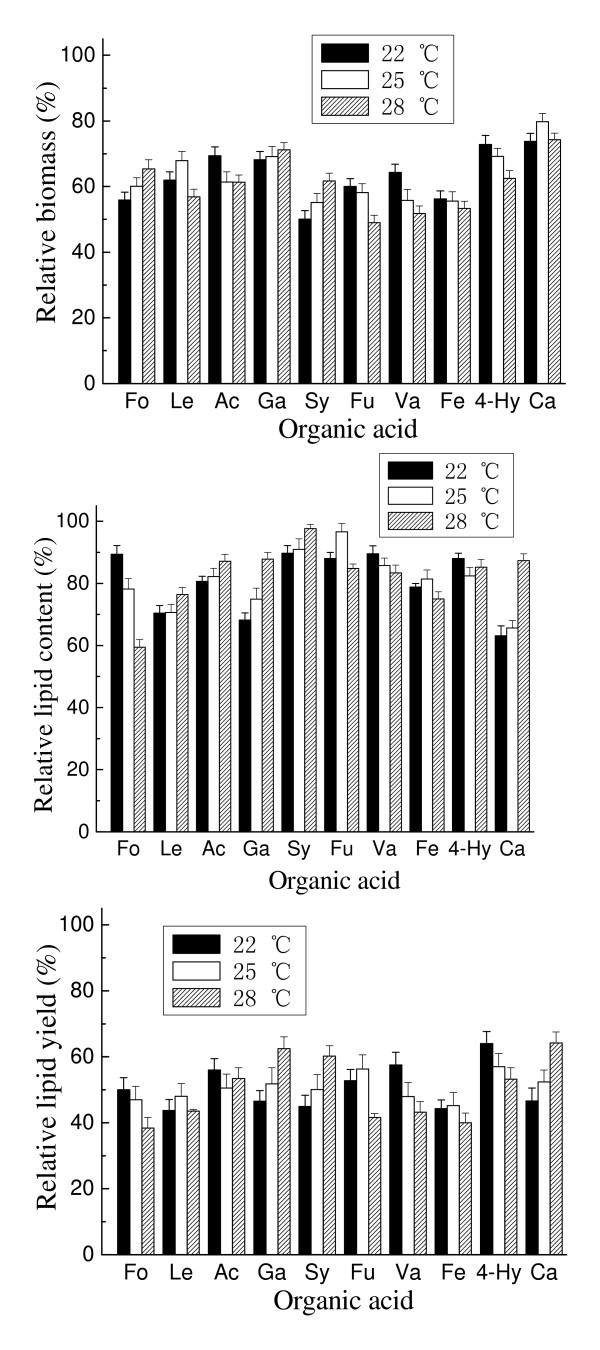
**Effect of temperature on the inhibition of organic acids**. All organic acids were tested at their respective molar concentrations of the tested organic acids that caused 50% inhibition of the lipid yield. Cultures with 5% inoculum size were incubated at initial pH 6.5 and 160 rpm for 7 days. Results are expressed relative to controls without organic acids. Biomass, lipid content and lipid yield of cultures lacking organic acids at 22°C, 25°C and 28°C were, respectively, 19.9 g/L, 55.8% and 11.1 g/L; 24.0 g/L, 61.7% and 14.8 g/L; and 23.6 g/L, 58.9% and 13.9 g/L. Ac, acetic acid; Fo, formic acid; Le, levulinic acid; Hy, 4-hydroxybenzoic acid; Fu, furoic acid; Ca, caproic acid; Ga, gallic acid; Fe, ferulic acid; Sy, syringic acid; Va, vanillic acid.

**Figure 8:**
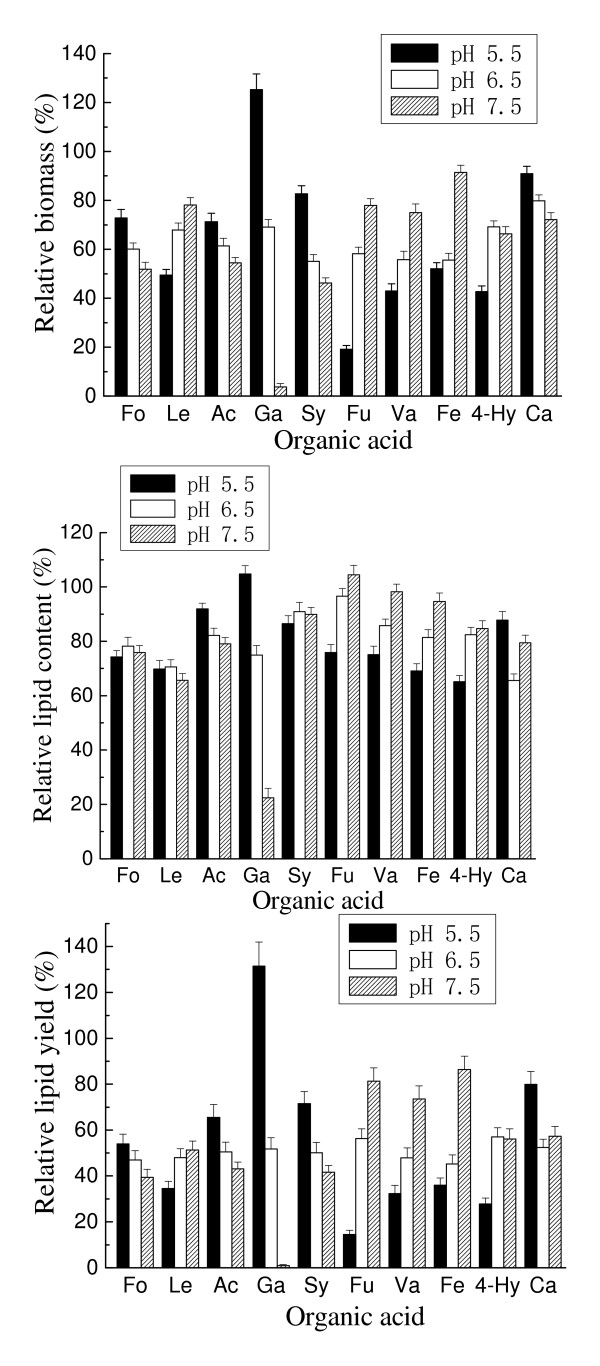
**Effect of initial pH on the inhibition of organic acids**. All organic acids were tested at their respective molar concentrations of the tested organic acids that caused 50% inhibition of the lipid yield. Cultures with 5% inoculum size were incubated at 25°C and 160 rpm for 7 days. Results are expressed relative to controls without organic acids. Biomass, lipid content and lipid yield of cultures lacking organic acids at pH 5.5, pH 6.5 and pH 7.5 were, respectively, 18.4 g/L, 57.2% and 10.5 g/L; 24.0 g/L, 61.7% and 14.8 g/L; and 21.5 g/L, 56.3% and 12.1 g/L. Ac, acetic acid; Fo, formic acid; Le, levulinic acid; Hy, 4-hydroxybenzoic acid; Fu, furoic acid; Ca, caproic acid; Ga, gallic acid; Fe, ferulic acid; Sy, syringic acid; Va, vanillic acid.

The effects of temperature and initial pH on the inhibition by organic acids at their respective IC_50 _concentrations were also recorded. No direct correlation between the culture temperature and the inhibitory effect of acids was observed (Figure 7). Similarly, there was no relationship between the initial pH and the inhibition by acids (Figure 8). Initial pH, however, exerted a greater impact than temperature on inhibition. Within the range of pH levels tested, for most of aliphatic acids, a higher initial pH resulted in a lower relative biomass. Interestingly, an opposite result was obtained in the presence of most aromatic or furanacids. It is worth noting that in some cases, a suitable pH can remarkably relieve and even eliminate the inhibitory effect of acids. For example, in the presence of gallic acid, the biomass and lipid content were only 0.8 g/L and 12.6%, respectively, at pH 7.5 (with corresponding relative biomass and lipid content being 3.8% and 22.4%). However, the toxicity of gallic acid dropped sharply with the decrease of pH and even an enhanced effect on cell growth and lipid accumulation was observed at pH 5.5, at which the biomass and lipid content reached as high as 23.1 g/L and 59.9%, respectively (with the relative biomass and lipid content being 125.3% and 104.8%).

### Effects of binary combinations of organic acids on cell growth and lipid accumulation of *T. fermentans*

It has been reported that the synergistic effect of different inhibitors present in the lignocellulosic hydrolysates is complex [32-34]. Therefore, the effects of binary combinations of organic acids on the cell growth and lipid accumulation of *T. fermentans *were tested at their respective IC_25 _concentrations listed in Table 2. Acetic and 4-hydroxybenzoic acids, the typical aliphatic and aromatic acids in lignocellulosic hydrolysate, respectively, were chosen for binary combinations with other organic acids. In the experiments, whenever two acids were combined, the predicted relative biomass, lipid content and lipid yield represented the values after deduction of the summed inhibition on biomass, lipid content and lipid yield by each of the two tested inhibitors at their IC_25 _concentrations. If the actual experimental value exceeded the predicted value, the inhibition was referred to as "synergistic."

**Figure 9:**
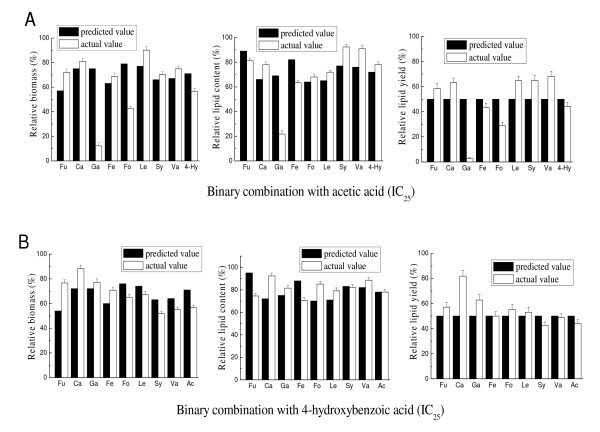
**Effect of binary combinations on the growth and lipid accumulation of *Trichosporon fermentans***. **(A) **Acetic acids (molar concentration of the tested organic acids that caused 25% inhibition of the lipid yield (IC_25_)). **(B) **4-hydroxybenzoic acid (IC_25_). Ac, acetic acid; Fo, formic acid; Le, levulinic acid; Hy, 4-hydroxybenzoic acid; Fu, furoic acid; Ca, caproic acid; Ga, gallic acid; Fe, ferulic acid; Sy, syringic acid; Va, vanillic acid.

As shown in Figure 9A, taking lipid yield into account for the binary combinations of acetic acid with other organic acids, the inhibition caused by most of the binary combinations was roughly equal to the predicted value. However, the combination of acetic acid with formic or gallic acid showed a synergistic inhibition on lipid yield. For example, the combination of acetic acid and gallic acid led to a 95% decrease in lipid yield compared to the predicted value. Likewise, in the cases of binary combinations of 4-hydroxybenzoic acid with other organic acids (Figure 9B), most of the binary combinations caused no synergistic inhibition. Interestingly, the binary combination of 4-hydroxybenzoic acid and caproic acid greatly reduced the inhibition on cell growth and lipid accumulation and resulted in a 60% increase in the lipid yield compared to the predicted value.

## Conclusions

The inhibitory effect of organic acids on the cell growth and lipid accumulation of *T. fermentans *can be relieved or even eliminated at low concentrations. Thus, more efforts should be made to improve the stress assistance of *T. fermentans *to inhibitors by genetic engineering and/or domestic methods. The development of cost-effective hydrolytic or detoxification processes to obtain lignocellulosic hydrolysates with lower inhibitor concentrations would also be useful. The inhibition of organic acids can also be reduced by optimizing culture conditions, such as inoculum size, temperature and initial pH of the medium.

## Methods

### Microorganism and chemicals

Oleaginous yeast *T. fermentans *CICC 1368 was obtained from the China Center of Industrial Culture Collection and kept on wort agar at 4°C. Levulinic acid, 4-hydroxybenzoic acid, syringic acid, vanillic acid and furoic acid were purchased from Alfa Aesar (Heysham, UK). Acetic acid, formic acid, caproic acid, ferulic acid, gallic acid and other chemicals were obtained from commercial sources and were of the highest purity available.

### Medium, precultivation and cultivation

The composition of the precultivation medium (pH 6.0) was as follows: glucose and xylose 20 g/L (ratio 2:1 wt/wt), 10 g/L peptone and 10 g/L yeast extract. The composition of the fermentation medium (pH 6.5) was as follows: 100 g/L glucose and xylose (ratio 2:1 wt/wt), 1.8 g/L peptone, 0.5 g/L yeast extract, 0.4 g/L MgSO_4_·7H_2_O, 2.0 g/L KH_2_PO_4_, 0.003 g/L MnSO_4_·H_2_O and 0.0001 g/L CuSO_4_·5H_2_O.

The preculture was performed in a 250-ml conical flask containing 50 ml of precultivation medium in a rotary shaker at 28°C and 160 rpm for 24 hours. Next, 5% seed culture (2.5 ml) was inoculated in a 250-ml conical flask containing 47.5 ml of fermentation medium, and cultivation was carried out in a rotary shaker at 25°C and 160 rpm for seven days.

### Effect of sugar concentration on the growth and lipid accumulation of *T. fermentans*

A mixture of glucose and xylose at a ratio of 2:1 (wt/wt) was used as the carbon source. The medium, at an initial sugar concentration of 25, 50, 75, 100, 125, 150, 200, 300 or 400 g/L, respectively, was used for the substrate inhibition study. After five days' fermentation, the biomass, lipid content, lipid yield and sugar concentration of *T. fermentans *on the medium with different initial sugar concentrations were compared.

### Effects of organic acids on the growth and lipid accumulation

After precultivation, 2.5 ml of seed culture were inoculated in 47.5 ml of fermentation medium containing the selected organic acid. To facilitate averaging, results throughout the text are expressed as percentages of the control values without addition of the tested inhibitor (with the biomass, lipid content, lipid yield and residual sugar concentration after seven days' fermentation being 24.0 g/L, 61.7%, 14.8 g/L and 15.7 g/L, respectively). IC_25 _and IC_50_, defined as the molar concentrations of the tested organic acids that cause 25% and 50% inhibition of the lipid yield of *T. fermentans*, respectively, were measured according to the data shown in Figure 3. All reported data were averages of experiments performed at least in triplicate.

### Effects of inoculum size, temperature and initial pH on the inhibition of organic acids

The effects of inoculum size, temperature or initial pH on the potency of organic acids were examined using acid concentrations of IC_50_. For inoculum size, 5%, 10% and 15% seed culture were inoculated in the fermentation media containing the selected acids (IC_50_). For temperature, the cultures with 5% inoculum size were maintained at 22°C, 25°C and 28°C, respectively. Fermentation media containing the assayed acids were adjusted to pH 5.5, 6.5 or 7.5 prior to inoculation to test the effect of initial pH. Biomass, lipid content and lipid yield were all measured after seven days' fermentation.

### Binary combinations of organic acids

Two selected organic acids with each concentration at IC_25 _were added to the fermentation medium. Cultures were inoculated as described above and incubated for seven days (5% inoculum size, pH 6.5, and 25°C). Cultures grown without adding organic acids were used as the control.

### Effects of organic acids on sugar utilization and malic enzyme activity

The effects of organic acids on sugar utilization were examined with the acid concentration being IC_25_. The relative sugar consumption was defined as the ratio of the amount of glucose and xylose consumed by the yeast cells grown on the culture medium containing the selected organic acid for seven days to that without the acid. The malic enzyme activity of *T. fermentans *was measured according to our previous work [25] with a SHIMADZU UV-2550 spectrophotometer (Kyoto, Japan).

### Analytical methods

Biomass was harvested by centrifugation and weighed in its lyophilized form [35]. Extraction of lipid from lyophilized biomass was performed according to a procedure modified from the one described by Folch *et al*. [36], with a mixture of chloroform and methanol (2:1 vol/vol). The extracted lipid was centrifuged to obtain a clear supernatant, and the solvent was removed by evaporation under a vacuum at 100 hPa, 55°C and 100 rpm (EYELA NE series rotary evaporator; Tokyo Rikakikai Co, Ltd, Tokyo, Japan). Lipid yield is expressed as the amount of lipid extracted from the cells in per liter of fermentation broth (g/L), and lipid content is defined as the percentage of lipid to dry biomass (% wt/wt). The fatty acid profile of the lipid from *T. fermentans *was determined by gas chromatography (GC-2010 Plus; Tokyo Rikakikai Co, Ltd) with an ionization detector and a DB-1 capillary column (0.25 cm × 30 m; Agilent Technologies Inc, Santa Clara, CA, USA) according to previously published procedures [10]. D-xylose and D-glucose were measured by high-performance liquid chromatography (Waters Corp, Milford, MA, USA) with a differential refractive index detector (Waters 2410; Waters Corp) and an Aminex HPX-87P column (300 mm × 7.8 mm; Bio-Rad Laboratories, Hercules, CA, USA) at 85°C. Deionized water was used as the mobile phase at 0.5 mL/minute.

## Abbreviations

IC_25 _and IC_50_: the molar concentrations of the tested organic acids that causes 25% and 50% inhibition on the lipid yield of *T. fermentans*; Con: Control trial on the medium without inhibitor; Ac: acetic acid; Fo: formic acid; Le: levulinic acid; Hy: 4-hydroxybenzoic acid; Fu: furoic acid; Ca: caproic acid; Ga: gallic acid; Fe: ferulic acid; Sy: syringic acid; Va: vanillic acid.

## Competing interests

The authors declare that they have no competing interests.

## Authors' contributions

CH designed and performed the experiments, analyzed the data and wrote the manuscript; HW designed the experiments, analyzed the data and revised the manuscript; ZJL performed several experiments, JC and WYL participated in the design of some experiments, MHZ coordinated the study and revised the manuscript. All authors read and approved the final manuscript.
